# Transport of ixodid ticks and tick-borne pathogens by migratory birds

**DOI:** 10.3389/fcimb.2013.00048

**Published:** 2013-09-10

**Authors:** Gunnar Hasle

**Affiliations:** Department of Biology, University of OsloBlindern, Norway

**Keywords:** ticks, migratory birds, tick-borne pathogens, tick-borne encephalitis virus, borrelia, babesia

## Abstract

Birds, particularly passerines, can be parasitized by Ixodid ticks, which may be infected with tick-borne pathogens, like *Borrelia* spp., *Babesia* spp., *Anaplasma, Rickettsia*/*Coxiella*, and tick-borne encephalitis virus. The prevalence of ticks on birds varies over years, season, locality and different bird species. The prevalence of ticks on different species depends mainly on the degree of feeding on the ground. In Europe, the *Turdus* spp., especially the blackbird, *Turdus merula*, appears to be most important for harboring ticks. Birds can easily cross barriers, like fences, mountains, glaciers, desserts and oceans, which would stop mammals, and they can move much faster than the wingless hosts. Birds can potentially transport tick-borne pathogens by transporting infected ticks, by being infected with tick-borne pathogens and transmit the pathogens to the ticks, and possibly act as hosts for transfer of pathogens between ticks through co-feeding. Knowledge of the bird migration routes and of the spatial distribution of tick species and tick-borne pathogens is crucial for understanding the possible impact of birds as spreaders of ticks and tick-borne pathogens. Successful colonization of new tick species or introduction of new tick-borne pathogens will depend on suitable climate, vegetation and hosts. Although it has never been demonstrated that a new tick species, or a new tick pathogen, actually has been established in a new locality after being seeded there by birds, evidence strongly suggests that this could occur.

## Introduction

There is ample evidence that birds, particularly passerines, can be parasitized by Ixodid ticks (Hoogstraal et al., 1961, 1963; Nuorteva and Hoogstraal, 1963; Anderson and Magnarelli, 1984; Mehl et al., 1984; Weisbrod and Johnson, 1989; Stafford et al., 1995; Olsen et al., 1995a; Nicholls and Callister, 1996; Smith et al., 1996; Ishiguro et al., 2000; Alekseev et al., 2001; Bjöersdorff et al., 2001; Scharf, 2004; Comstedt et al., 2006; Poupon et al., 2006; Ogden et al., 2008; Hasle et al. 2009). These ticks may be infected with tick-borne pathogens, like *Borrelia* spp. (Olsen et al., 1995a,b; Gylfe et al., 2000; Hanincova et al., 2003; Comstedt et al., 2006; Poupon et al., 2006; Ogden et al., 2008; Hasle et al. 2010; Kjelland et al., 2010; Franke et al., 2012; Socolovschi et al., 2012), *Anaplasma* spp. (Alekseev et al., 2001; Bjöersdorff et al., 2001; Daniels et al., 2002; Ogden et al., 2008; Franke et al., 2012), *Babesia* spp. (Hasle et al., 2011), *Rickettsia*/*Coxiella* (Elfving et al., 2010; Socolovschi et al., 2012) and Tick-borne encephalitis virus (TBEV) (Waldenström et al., 2007; Geller et al., 2013). The prevalence of ticks on birds varies between years, season, locality and different bird species. The prevalence of ticks on different species depends mainly on the degree of feeding on the ground (Mehl et al., 1984; Hasle et al. 2009; Marsot et al., 2012). In particular, thrushes, i.e., the *Turdus* spp. in Europe (Hasle et al. 2009), Russia (Alekseev et al., 2001) and Japan (Ishiguro et al., 2000), and *Catharus* spp. in North America (Smith et al., 1996), have a high prevalence of tick infestation. e.g., in our Norwegian material 31.5% of blackbirds, *Turdus merula*, and 25.1% of song thrushes, *T. philomelos*, were infested by ticks (Hasle et al. 2009). Blackbirds are often infected with several ticks on each bird. Five per cent of blackbirds (*N* = 543) had ten or more ticks, and one single blackbird had 66 nymphs of *Ixodes ricinus* (own data). Only one study has compared historic and new data concerning ticks on birds. Hasle et al. (2009) compared data from 2003 to 2005 with Mehl et al.'s (1984) data from 1965 to 1970 on two Norwegian bird observatories, and found an increase of the prevalence of tick infestation on passerine northward migrating birds from 4.2 to 6.9% (*p* < 0.001). Birds can easily cross barriers, like fences, mountains, glaciers, desserts and oceans, which would stop mammals, and they can move much faster than the wingless hosts. Therefore, birds have a potential of spreading ticks far beyond the home ranges of mammals and reptiles. Migrating bats can, like birds, cross barriers and move long distances in a short time, and could also have a potential of transporting ticks and tick-borne pathogens, like *Bartonella*, *Borrelia* spp. and members of the family Rickettsiales (Gill et al., 2008; Mühldorfer, 2013).

## Introducing new tick species

**Figure 1:**
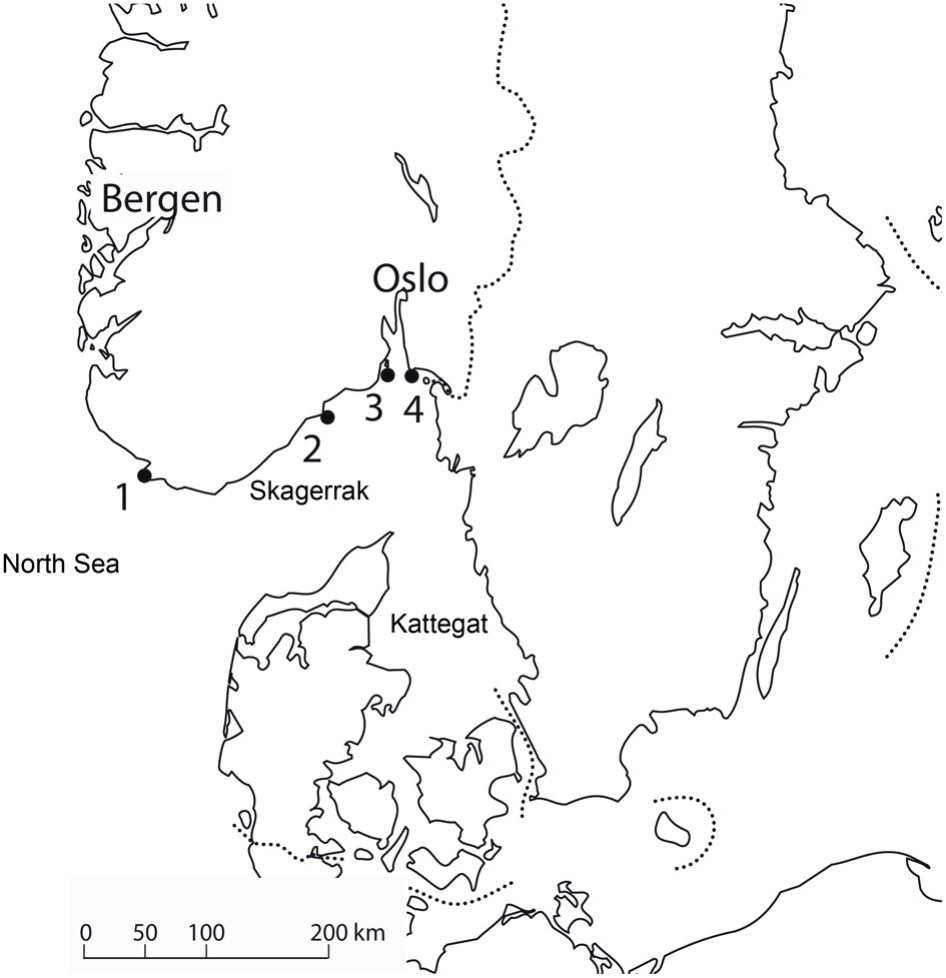
**Study area of Hasle et al. (2009).** The participating bird observatories were: (1). Lista, (2). Jomfruland, (3). Store Færder, (4). Akerøya. Northward migrating birds would have to cross The North Sea, Skagerrak or Kattegat.

Although it has never been demonstrated that a new tick species, or a new tick pathogen, actually has been established in a new locality after being seeded there by birds, the evidence strongly suggests that this could happen. Hasle et al. (2009) studied northward migrating birds on four bird observatories situated north of the Skagerrak and Kattegat (Figure 1). From 9.768 passerine birds examined they found seven nymphs of *Hyalomma rufipes*. The *Hyalomma* species have a northern distribution limit in Southern Europe and North Africa (Estrada-Peña et al., 2004), and these nymphs would have had to be transported all the way from the Mediterranean to Norway, the last 130–150 km over open sea. Although many *Hyalomma* individuals may be brought to Norway every year, they would not settle in a cold temperate climate. Also, a larva of *Dermacentor* sp. was found on a willow warbler, *Phylloscopus trochilus* on Akerøya, Hvaler, an island off the Southern Norwegian coast. No *Dermacentor* sp. is endemic in Norway. These findings demonstrate that non-endemic tick species can be transported to new places, across geographic barriers. Considering an influx of 30–80 million passerines crossing the sea every spring, millions of ticks will be transported every year, and transport of exotic species would not be a rare event. The limiting factor will not be the ticks' dispersal ability, but the suitability of the area the ticks are released for the survival and reproduction of the ticks. A suitable climate is one of the prerequisites for establishing a tick species. For *I. ricinus*, which has its northern border of distribution in Norway and Sweden, the distribution correlates well with the isolines for the first frost night, number of days with snow cower, and the length of the growth season (Jaenson et al., 2009). As these isolines overlap we cannot from these data tell which climatic factor is the most important. As ticks are prone to desiccation (Daniel and Dusbábek, 1994), microclimatic conditions of sun, soil and vegetation cover will determine the individual ticks' chance of survival, much like in the Parable of the Sower (Mattew 13:5). *Dermacentor reticulatus*, which occurs in North Germany, is a vector for *Babesia canis*, *F. tularensis*, *Rickettsia slovaka* and *C. Burnetii*. It needs to complete the whole life cycle during one year (Dautel et al., 2006), which unlikely could take place during a normal year in Norway. However, future climate change could make Norway hospitable for this species. Conceivably, individual specimens of *D. reticultus* could transmit diseases after being transported to Norway by birds. An indirect evidence of this is a case of *Babesia canis* in a Norwegian dog that had not been abroad. *D. reticulatus* is the main vector for *B. canis* (Øines et al., 2010).

Apart from protection from frost and desiccation, a good tick habitat will depend on appropriate hosts. e.g., islands that harbor only small animals cannot support the full life cycle of *I. ricinus*, as the adults need hosts of a certain minimum size (Jaenson et al., 1994). Possibly, some tick species may be host specific, although this is controversial (Randolph, 2004).

For establishing a new population, an ecological *niche* must be available. According to the Competitive exclusion principle, two different species cannot sustainably occupy the same *niche* (Hardin, 1960). However, this is a theoretical consideration, and very similar species, like *I. ricinus* and *I. persulcatus* may have different climate requirements, e.g., *I. persulcatus* is much more cold-resistant than *I. ricinus* (Tokarevich et al., 2011). Therefore, their *niches* are not exactly identical. Furthermore, in a transient situation sympatric distribution can occur, as is shown in a recent publication showing a shift in the distribution range of *I. persulcatus* toward North and West in Karelen, Russia, into areas previously occupied by *I. ricinus* (Bugmyrin et al., 2013). It is shown for *I. persulcatus* (Filippova, 2002) as well as *I. scapularis* (Oliver et al., 1993) that they can readily mate and breed with *I. ricinus*, but the offspring is sterile. In a situation with coexistence, the reproductive success of the least abundant species would suffer much more than the most abundant species (Hasle, 2010). Ticks seeded in an area occupied by another species with which they can interbreed would have a very low chance of copulating with the same species.

Climate change, as well as changes in vegetation, is expected to influence the distribution of different tick species (Estrada-Peña, 2001; Gray et al., 2009). Global warming would be expected to push a Northern limit of a distribution range further north. Such changes take place over decades, and dispersal by mammalian tick-hosts would be sufficient to keep up with the changes within a continent. However, if a geographical barrier, e.g., a sea, like Skagerrak and Kattegat, separates two hospitable areas, one with a tick species, and one without that species, and if the ecological *niche* is not occupied by another tick species, transport by birds would be a possible mechanism.

## Transport of tick-borne pathogens

Birds can potentially transport tick-borne pathogens by transport of infected ticks. In addition, the birds may act as amplifying host by being infected with tick-borne pathogens that can infect their parasitizing ticks, which can transmit the pathogens to subsequent hosts, or by hosting co-feeding ticks, i.e., ticks that feed near each other on the same host, when pathogens may pass from one tick to the other.

### Transport of infected ticks

Transport of already infected ticks is an obvious mechanism of transporting tick-borne pathogens by birds, as most tick-borne pathogens survive transstadially, i.e., from larvae to nymphs, and from nymphs to adults. This is shown for *Anaplasma marginale* (Stich et al., 1989), *Babesia divergens* (Bonnet et al., 2007), *Borrelia burgdorferi* sensu lato (Bellet-Edimo et al., 2005) and TBEV, and is indeed a prerequisite for being a tick-borne pathogen (Randolph, 2011). However, the transstadial survival may not be 100%, as some data indicate that *B. afzelii* can disappear from *I. ricinus* (Matuschka and Spielman, 1992) and *B. burgdorferi* s.s. from *I. scapularis* (Ogden et al., 2008) that are feeding on birds in Europe and North America respectively. Corresponding to this, Hasle et al. (2010) found that engorged ticks had a lower prevalence, i.e., odds ratio 0.24 (*P* = 0.004), of *B. afzelii* than unengorged ticks from birds. To spread a pathogen, a later stadium of the tick would have to parasitize a host that can mediate further transmission. Adult *I. ricinus* parasitize animals of the size of cats and larger (Jaenson et al., 1994), while small rodents and birds are the main reservoirs for the *Borrelia* spp. most common in Europe (Kurtenbach et al., 2002). Therefore, when fully engorged, *Borrelia*-infected nymphs molt to adult stage, it is not obvious that they can spread the pathogen further. However, although not very efficient, transovarial transmission is described for Babesia (Bonnet et al., 2007), Borrelia (Bellet-Edimo et al., 2005) and TBEV (Danielová and Holubová, 1991), which means that infected larvae can be transported by birds, and then molt to infected nymphs. Likewise, adult ticks may produce infected offspring after arrival on a new place, by transovarial transmission.

Human pathogens can also be transported by ticks that normally don't bite humans, as is shown for *I. uriae* (Olsen et al., 1995b).

In the case of TBEV, the virus is thought to be maintained in nature mainly by transmission through co-feeding on small rodents, as infected nymphs spread the virus to co-feeding larvae (Labuda and Randolph, 1999). It is almost exclusively nymphs and larvae of *I. ricinus* that can be transported by birds (Jaenson et al., 1994; Hasle et al. 2009). As the bird host would be the first host for the feeding larvae, they would not carry TBEV before entering the bird unless the virus is transmitted transovarially. Infected nymphs would proceed to adult stage after feeding, and would not feed on small rodents. Therefore, the TBEV-infected ticks transported by birds would be a dead-end for maintaining the TBEV in nature, without transovarial transmission. Assuming transovarial transmission, the TBEV could be seeded by birds to new areas if the climate, vegetation and hosts are suitable for the natural TBEV-cycle. In Norway, the first cases of TBE were notified in Southern Norway in 1998. Since then there has been an increase in annual cases, to the present 10–15 cases per year. A very relevant question would be why this has not happened before. The answer may well be that it has happened before, but that the conditions necessary for maintaining the fragile cycle of TBEV (Randolph and Rogers, 2000) in the nature have not been present before. The distribution of TBEV in Scandinavia is discontinuous, with at least 300 km of land distance from the endemic areas in Sweden (Båhuslän) to the Agder counties in Norway, while crossing the Skagerrak or Kattegat sea is a three to five hours flight for small passerine birds. It is difficult to find another explanation for this discontinuous distribution than transport via birds. This is the closest we can come to a proof that birds have seeded a tick-borne infection to a new area.

*Babesia venatorum* is an emerging tick-borne human disease in Europe. The first findings of this parasite in Norway have been on four nymphs of *Ixodes ricinus* brought to Norway by birds (Hasle et al., 2011). *B. venatorum* is a primarily a roe deer parasite (Duh et al., 2005), and adult *I. ricinus* readily feed on roe deer. Therefore, birds could effectively import and spread this parasite.

### Birds infected by tick-borne pathogens

Birds can be infected by different genuses of Anaplasmataceae, i.e., *Anaplasma phagocytophilum* (de la Fuente et al., 2005), *Rickettsia rickettsia* (Lundgren et al., 1966) and *Coxiella burneti* (Babudieri and Moscovici, 1952). We have not found any experimental data confirming that birds infected by the Anaplasmataceae can transfer these pathogens to ticks. The *Babesia* spp. appears to be host specific, at least to the class, and some of them to one species (Peirce, 2000), no mammal *Babesia* sp. has been found infecting birds. Some species of *Borrelia* can infect birds, notably *B. garinii*, *B. valaisiana*, *B. turdi* (Gylfe et al., 2000; Richter et al., 2000) and *B. burgdorferi* s.s. (Anderson and Magnarelli, 1984; Anderson et al., 1986), but probably not *B. afzelii* (Kurtenbach et al., 2002), although this notion has been challenged by the findings of Franke et al. (2010). Hasle et al. (2010) found an odds ratio for *Borrelia* infection of 4.3 for ticks parasitizing the *Turdus* spp., i.e. 3.5 for *B. garinii* and 30.3 for *B. valaisiana*. This indicates that the *Turdus* spp. are more susceptible for infection with these *Borrelia* genospecies than other birds, and that the ticks got the pathogen during feeding on the bird. Thus, the *Turdus* spp., apart from being likely to carry ticks, were more likely to carry *Borrelia*-infected ticks than other bird genera. Therefore, the *Turdus* spp., especially the blackbird, may be important in the spreading and hosting of *B. garinii*, which is the main agent for neuroborreliosis (van Dam et al., 1993; Balmelli and Piffaretti, 1995).

The *Borrelia* species causing tick-borne relapsing fever are transmitted by soft ticks, *Ornithodorus* spp., which have a different biology from the *I. ricinus*-like ticks in that they live in nests and burrows and have a feeding time of just a few minutes Johnson and Golighty (2000). These ticks would not be prone to long-range transport by birds, but the birds may contract the *Borrelia*, which can be transmitted further, as suggested for *Borrelia hermsii*, by (Schwan et al., 2007).

Transport of tick-borne pathogens from one endemic area to another could have an impact, even if the pathogens already occur there, by spreading new strains to new areas.

### Transfer of pathogens through co-feeding

Transmission of a tick-borne pathogen through co-feeding has been demonstrated for the tick-borne encephalitis-virus (TBEV) on *Myodes glareolus* and *Apodemus sylvaticus* (Labuda and Randolph, 1999), but no data exists for such transmission of TBEV on birds. In Waldenström et al.'s material (2007) they found three TBEV-positive *I. ricinius*, one nymph and two larvae, on one individual European robin, *Erithacus rubecula*, which strongly suggests that the bird either was viremic or that transmission through co-feeding did occur.

Also, for *Borrelia* this mechanism has been suggested for mammals. Gern and Rais (1996) demonstrated transmission of *B. burgdorferi* between co-feeding *I. ricinus* on AKR/N mice in the laboratory. By using a generalized linear model (GLM) Hasle et al. (2010) found that ticks that were co-feeding on birds with ticks infected with one genospecies of *Borrelia* had an increased probability of being infected with the same genospecies. A tick co-feeding with another tick with *B. afzelii* had an odds ratio of 3.9. If the statement that birds cannot be infected with *B. afzelii* is correct, this is an indication that non-systemic transmission between co-feeding ticks has occurred. For *B. valaisiana* the odds ratio for ticks co-feeding with other ticks with *B. valaisiana* was 760, compared to the average prevalence. The latter could partly be explained by the effect of feeding on the *Turdus* spp., and by systemic infection of the bird. Geller et al. (2013) found the same strain of *B. afzelii* (PGau) on two *I. ricinus* nymphs that were feeding on the same Great tit (*Parus major*), a finding that suggests transmission through co-feeding.

The existing data support the hypothesis that all the three mechanisms of spreading tick-borne pathogens by birds may occur in the nature.

## Migration routes

Knowledge of the bird migration routes is crucial for understanding the possible impact of birds as spreaders of ticks and tick-borne pathogens. For instance, the main direction of migration in Europe is NE/SW for the *Turdus* spp., *E. rubecula, Phoenicurus phoenicurus, Prunella modularis* and *Troglodytes troglodytes* (Gjershaug et al., 1994; Bruderer, 1997; Fransson and Hall-Karlsson, 2008), which are all species with a high prevalence of tick infestation (Hasle et al. 2009; Marsot et al., 2012). These species would be unlikely to transport the TBEV from Central to North-West Europe, but could possibly transport this virus from Central to South-West Europe. On the other hand, species that migrate along a more N/S direction across the Alps, like *Sylvia curruca, S. communis* and *S. atricapilla*, or NW/SE, like *Motacilla alba* and *Luscinia svecica* (Gjershaug et al., 1994; Fransson and Hall-Karlsson, 2008) could potentially cross the Skagerrak sea and transport ticks carrying TBEV from the TBE-endemic areas in Central Europe to Norway and Sweden. During NW migration *Motacilla alba* and *Luscinia svecica* may pass areas endemic for *Ixodes persulcatus* before reaching Sweden, and could, theoretically, introduce this tick species to Sweden, as the climate in Sweden would be suitable for *I. persulcatus*. However, the ecological *niche* is already occupied, by *I. ricinus*, and *I. persulcatus* would therefore be unlikely to colonize Sweden, even if they were brought there by birds. On the other hand, to bring this speculation further, if they can be transported past areas occupied by *I. ricinus* it could still remain a possibility for colonization of *I. persulcatus* in Sweden. *I. persulcatus* survives extreme winter temperatures in Siberia, and could possibly do that in Scandinavia as well. The findings of *I. persulcatus* and human cases with S-TBEV in Kokkola (N63°50′ E23°07′), Finland, several hundred kilometres from the known western distribution range of *I. persulcatus* (Jääskeläinen et al., 2006), suggests long distance transport by birds.

## Conclusions

Transport of ticks by migratory birds, including tick species and tick-borne pathogens that are not endemic to a new area, is a common event. Establishment of a new tick species in an area will require favorable climate, vegetation and hosts, and an available ecological *niche*. New tick-borne pathogens could be spread if there are susceptible tick and vertebrates hosts present. It still remains to prove if this way of dispersal has happened.

### Conflict of interest statement

The author declares that the research was conducted in the absence of any commercial or financial relationships that could be construed as a potential conflict of interest.
